# Seen but not heard: A qualitative interview study exploring the views and experiences of children and young people referred to Child and Adolescent Mental Health Services for support with suicidality

**DOI:** 10.1371/journal.pmen.0000539

**Published:** 2026-06-30

**Authors:** Lynne Gilmour, Margaret Maxwell, Edward Duncan

**Affiliations:** Centre for Healthcare and Community Research, University of Stirling, Stirling, United Kingdom; Uganda Martyrs University, UGANDA

## Abstract

Suicide is a leading cause of death amongst children and young people (CYP), and numbers of CYP presenting with suicidality continue to rise. CYP seeking help for suicidal thoughts and behaviours are generally referred to Child and Adolescent Mental Health Services (CAMHS) for assessment and treatment. However, CAMHS across the UK are unable to meet the demand for their services. Little is known about what happens to these children after they have been referred to CAMHS.

Grounded in critical realism, and informed by a children’s rights approach and feminist perspectives, this qualitative study sought to explore how children and young people presenting with suicidality experience the CAMHS referral process and care journey thereafter. In-depth interviews of between 60–90 minutes duration, were conducted with ten CYP aged between 13–17yrs, living in two different health board areas in Scotland, and having been referred to two different CAMHS (sites A and B).

Using Charmaz constructing grounded theory approach, analysis revealed three main themes which are presented: Nothing got resolved: the care experience; “If you had more choice…” about how, when and where they worked with you; The person not the profession who helped, each built upon layers of overlapping subthemes. Further interrogation of the data, and synthesis supported the development of a substantive theory, that conceptualises children and young people presenting with suicidality as “Seen but not Heard”. It is argued that CAMHS do not meet these CYP needs even when they are seen.

This finding has implications for policy makers and service providers as the CYP in this study express that the support they need and find most helpful is not congruent with CAMHS. Further research is urgently required to develop and design a new service model that better meets the needs of children and young people and prioritises their views.

## Introduction

Suicide is the third leading cause of death amongst children and young people (CYP) globally [1] and the leading cause in the UK. Addressing this is a priority for many governments including the UK and devolved nations’ Governments [2–4]. Children (aged <18 years) seeking help with suicidality (thoughts and/or behaviours) are generally referred to Child and Adolescent Mental Health Services (CAMHS) for assessment and treatment. However, CAMHS across the UK are unable to meet demand for their services [5–7]. A recent study in Scotland found that 25% of referrals to CAMHS are for CYP experiencing suicidality [8]. A survey of 13,887 of CYP in the UK found that not only does the mental health of young people deteriorate during long waiting times, 26% of respondents had attempted suicide while waiting for mental health support [9]. CAMHS is often considered the best and often only option for CYP experiencing suicidality, and yet little is known about how CYP experience the process of being referred to CAMHS, or the support they receive if and when they are seen.

Most previous qualitative research exploring CYP experiences and CAMHS is not specific to those who have experienced suicidality [10,11], or excludes CYP who have been or are suicidal [12–14]). Findings from related studies of CYP views of CAMHS services in general, suggest CYP face many barriers to accessing CAMHS, and that the treatment and support they receive is often found to be lacking [15–17]. Notably, one recent qualitative analysis of Tik Tok videos posted by young people relating to CAMHS found that young people did not feel their distress was addressed through engaging with CAMHS, although again the sample did not all have specific experience of help seeking for suicidality [18]. Furthermore, although participatory approaches are now more common in mental health research and service design, the voices of CYP with experience of suicidality often remain absent, despite the urgent need to address their growing numbers

This paper presents the findings from a series of qualitative interviews with children and young people who were referred to CAMHS for suicidal ideation or a suicide attempt in two different regions of Scotland (Site A and B) between 2019–2020, revealing that even when they are seen by CAMHS the type of treatment and support received does not meet their needs. This study was conducted as part of a larger mixed methods study which also established the pathways of care for CYP referred to CAMHS for suicidality, as well as the views of parents/carers and practitioners of this care journey [14]. The research question we sought to answer with this qualitative study was: *How do children who are referred to CAMHS for suicidality experience their treatment and journey of care?* We aimed to explore children’s experiences of being referred to CAMHS for suicidality and to present their care journey from their perspective.

## Methods

### Ethics statement

Approvals for this study were granted by the University of Stirling NHS, Invasive or Clinical Research Ethics committee, NHS North of Scotland Research Ethics Committee (Reference No: 19/NS/0031), as well as the adjacent Research & Development offices and Caldicott guardians with responsibility for the site regions. Written informed consent was gained form all participants prior to interview, parental consent was not necessary as all participants were aged 12yrs> (see Ethical considerations below).

### Study design

This was a qualitative interview study employing a constructivist approach, to interview children and young people who had been referred to CAMHS primarily for reasons of suicidality, about their experiences of seeking help from CAMHS.

### Data collection

Semi-structured interviews were chosen to collect data, as they provided the most appropriate form of data to answer the research question, as well as promoting the safety and confidentiality of participants that may have been compromised by other qualitative methods such as focus groups. Interviews allow participants voices to be heard [19], reflecting a children’s rights-based approach, and feminist position which has primarily been concerned with ‘giving voice’ to those from marginalised or disenfranchised communities. Qualitative interviews also support a phenomenological approach concerned with the individuals lived experience [20] which can also then be interpreted according to theoretical understandings and beliefs about how the meaning attached to these experiences is constructed. Thus, they were best suited to answer our research question which was concerned with experience and perspective of CYP.

Essentially, interviews are a conversation. The dialogue between interviewer and participant co-produces new knowledge [21]. However, conversations in the context of a research interview are subject to pronounced power differentials, and directed rather than free forming, requiring significant reflexivity on the part of the researcher in relation to their presentation and interpretation of what is said [22]. Therefore, although the research attempts to ‘give voice’ to the experience of CYP, as in any qualitative research what is presented is the interpretation of the researchers, influenced by their own background and beliefs [23].

### Data analysis

We used Charmaz’ [24] ‘Constructing Grounded Theory’ to analyse the interview data. This approach recognises the existence of multiple realities, and the role of the researcher in constructing an interpretation of those presented by the participants. It also allows for the researcher’s theoretical position to be recognised alongside fluidity in data collection as it is informed by and evolves from the analysis [24]. For example, it acknowledges each interview is not conducted in entirely the same way, and as they progress, questions can be asked to support testing out ideas that emerged from previous interviews. It concentrates on eliciting rich interview data allowing for in-depth analysis, rather than the number of participants. Memo writing, and early analysis was undertaken alongside data collection to help inform the development of early codes.

Interview transcripts were initially coded line by line in NVivo 11. Further focused codes were developed following an iterative process of constant comparison, and reflection and the creation of concept maps. Standing back from the data and using story-line analysis helped to explain the process and refine these focused codes and grounded theory ‘into a digestible format’ [25]. Theoretical coding emerged from this process, reflecting the wider knowledge accrued through the research journey, analysing and identify relationships between codes [24]. Thus, the findings from this study go beyond describing what was said, to offer an overall argument. As with all qualitative research, what we present is our interpretation of the data, other researchers may have approached the issue differently and interpreted the findings in a different way. Acknowledging this and being both reflexive and transparent in reporting provides an understanding of how conclusions were reached.

LG led the analysis, with MM and ED conducting initial independent coding of two transcripts prior to the code book being finalised and thereafter meeting to discuss concept maps and thematic codes as they developed.

#### PPI: Involving children and young people.

Research has shown that visual imagery and multi-media formats can improve acceptability and understanding of the information presented to children [26–28]. Recruitment materials were designed by young people at the MacRobert Arts Centre Film Crew and Graphic Media Group (both groups were for CYP aged 13–17 yrs, there were 6 CYP in the Film Crew, 2 were female, and 5 in the Graphic Media Group, 2 were male). The Film Crew created a short animation to support the informed consent process by explaining in a visual and accessible format what the research study was about and what was being asked of participants. Young people in the Graphic Media Group had heard about the project and asked the researcher if they could design posters and postcards to support recruitment. All young people were given vouchers to thank them for their time, and a special event was organised to launch the animation and postcards at the MacRobert Arts Filmhouse to an audience of invited guests, with the CYP showcasing their work.

Interview topic guides (S1 File) and information leaflets were reviewed by CYP volunteers and resource workers at ‘SEE ME’, a national organisation in Scotland tackling mental health stigma. They were also provided with vouchers as a token of thanks for their time.

#### Sample and recruitment.

Constructivist grounded theory was also selected as it does not require sampling until data saturation is reached as is the case with a traditional grounded theory approach [24,29]. Although there are many different views upon how many interviews are enough, six in-depth interviews have been suggested by Guest(2013) as ‘The magic number’ [29]. The rationale being that the majority of coding, even in much larger sample populations, occurs within the first six interviews and is also supported by the recommendations of others [30]. The number of participants is believed to be less important than whether the interviews are ‘informationally representative’ [31], that is, the data provides a rich, in-depth understanding of the phenomenon being researched.

Debates about the ideal qualitative sample size continue, however, it is broadly accepted that judgements need to be made by researchers [30,31]. A recent systematic review of sample sizes in qualitative studies found that reporting of sample size was often lacking, or unclear [32]. As is often the case with qualitative methods debates much depends upon the epistemological position of the researcher as much as external constraints such as funding requirements etc. Pragmatic considerations about what was achievable with the resources and time available, influenced decision-making around sample size.

We aimed to recruit approximately twelve children and young people aged 8–18 who had been referred to CAMHS in either site A, or site B, for reasons of suicidality during a six-month period (Jan – June 2019 inclusive). Eligible participants were identified via a retrospective cohort study in these sites, whereby all referrals made over this six-month period were screened to identify those where the primary reason for referral had been suicidal thoughts and/or behaviour [8]. We sought to recruit even numbers of participants in relation to gender, age, site, and those whose referral had been rejected/ put on waiting list, and those seen by CAMHS. However, recruitment was challenging and as much as we adopted a purposive sampling strategy it was also convenience sampling (participants self-selected by opting in), and our focus was upon ensuring it was “*informationally representative*” [31]. This was achieved through conducting high quality, probing, and comprehensive interviews whereby participants shared rich, in-depth insights about their experience.

We excluded anyone under 8yrs or over 18yrs of age, or where the primary reason for referral to CAMHS was something other than suicidality, or if they were hospitalised as an in-patient at the time of recruitment.

Potential participants from a sampling frame of n = 214 were identified from the retrospective cohort arm of the study [8]. An initial invitation to participate, and recruitment pack was sent from the National Health Service (NHS) CAMHS site, introducing the researcher, inviting them to get in touch if they wished to find out more about participating. The recruitment pack contained a letter, participant information sheet, postcard with a QR code link to a study web-site, and a graphic business card with flash drive built in that played an animation. Invitations were titrated (20 at a time in site A, and 40 in Site B due to slower recruitment) to avoid over recruitment of children and young people who would not then be invited to interview. An information pack and invitation to participate in the parent interview arm of the study [14] was sent to parents/guardians at the same time as the child/young person. If the child was over 12 years of age and preferred not to inform their parents they were interested in participating, their right to privacy was respected, but they were also encouraged to identify a safe adult, e.g., teacher or CAMHS worker (if they are involved with CAMHS) they were happy to share this intention with who they would be happy for the researcher to contact prior to interview and if there were any safety concerns that arose during interview. Where children were too young to give consent (under 12 years), parental consent was to be sought. CYP and/or their parents could get in touch by phone, text, email or a web-site contact form.

### The interviews

Interviews were offered to take place wherever was most convenient and safe for the CYP (e.g., home, school, a community venue) and could be conducted face to face or by telephone. They could also choose for someone to be with them during the interview, e.g., a family member/ teacher/ support worker or friend. The researcher provided a range of resources to support engagement in a safe, relaxed way, e.g., modelling clay, fidget toys, arts and craft materials. Following the imposition of COVID 19 lockdown restrictions it was necessary to conduct the final few interviews via Skype as meeting in person was prohibited. Prior to these interviews, several telephone conversations were often required with CYP and parents to ensure they fully understood the consent process and what was expected in relation to their participation.

Interviews were conducted between October 2019 – March 2020. All interviews were audio recorded and transcribed verbatim by an independent transcriber with whom the University has a data sharing agreement.

All participants were provided a voucher as a token of thanks for their time, and travel expenses were paid where required.

### Ethical considerations

Children are deemed a vulnerable group because adults have power over them [33]. Research involving children is understandably subject to high levels of ethical scrutiny. Researching children’s lives and the issues faced by especially vulnerable children (such as those who have attempted suicide) is often unaddressed [34]. Recent depictions of children as autonomous agents [35], supported by the children’s rights agenda, promote the position that children should be consulted in research; they have a right to be heard, listened to and consulted on matters that affect them [36,37]. The UNCRC (1989) has supported development of high international ethical standards within UK legislation [38].

There is evidence to show the benefits to children who participate in research, and this includes groups of vulnerable children for whom the research process gives power to their voice, validates their experience, and allows them to be heard [37,39]. Children who present with suicidality are considered especially vulnerable and often excluded from research trials for fear of risk [40,41]. However, there is an emerging body of evidence suggesting that contrary to concerns that being involved in research may cause harm to participants, the experience of being involved in qualitative research for suicidal young people can have a positive impact on their well-being [42–44]. A recent literature review found there to be no evidence to support the most prominent fear that talking to participants about suicide will make them more vulnerable to suicide; in fact, they found it may help to improve mental health [42].

Given the nature of the research topic, there was a risk that participants could become distressed during the interview, or immediately after. Distress protocols [45] were implemented that included creating emotional safety plans with people prior to the interview. These set out clear actions which would be taken should there be a disclosure of a child protection nature, and or an expression of suicidal intent. CYP were made fully aware as part of the informed consent process what would happen with their information, and assured that what they shared during the interview would be held confidentially unless they or someone else was in immediate danger or at risk of harm. At the end of the interview, all participants were given an information sheet of local resources and national helplines and encouraged to identify a person they would speak to if they were upset. LG’s previous experience of supporting children and young people who have been suicidal, and their families, equipped them with the knowledge and understanding to ensure their responses were sensitive, appropriate and informed.

Researcher emotional safety was also considered throughout, with a co-author available for de-brief after every interview.

### Reflexivity

In this study, we adopt a critical realist approach as our theoretical position. Critical realism supports the view that an independent reality exists, but also that multiple interpretations of and versions of realities may also exist [46]. All interviews were conducted by the first author (LG) who has around 20yrs experience of working in various roles and contexts within front line services (third sector) with CYP who presented with suicidal thoughts and behaviours, as well as experience of conducting research with CYP experiencing suicidality and conducting research with other vulnerable and marginalised groups. LG’s lived professional experience of young people being referred to CAMHS for suicidality was that they seemed to have a variety of different responses and experiences, and the lack of research around this was a rationale for the study. Second author MM, and third author ED also have many years of experience in conducting and managing qualitative mental health research projects.

## Results

### Sample population & dataset

Ten interviews were conducted with participants aged 13–17 years in Site A and B, seven of which identified as female, all were white British. Interviews took place within the child or young person’s home (n = 5), community centre (n = 1), place of education (n = 3) and following the COVID -19 lockdown via skype (n = 2). Two of the young people requested their parent be present throughout their face-to-face interview, resulting in these being joint interviews with the parents.

Three of the children lived in Site A, all the other participants lived in Site B. Three of the children lived in single parent households, one lived with a parent and step-parent, the remaining six lived at home with both parents. Nine participants were of school age, seven attended school, one attended a local authority ‘alternative to school’ provision, and one child did not go to school. One participant was attending University, although they had been in high school at the time of their referral to CAMHS. At the time of interview only one participant was actively involved with CAMHS, and one was on the waiting list.

**Fig 1:**
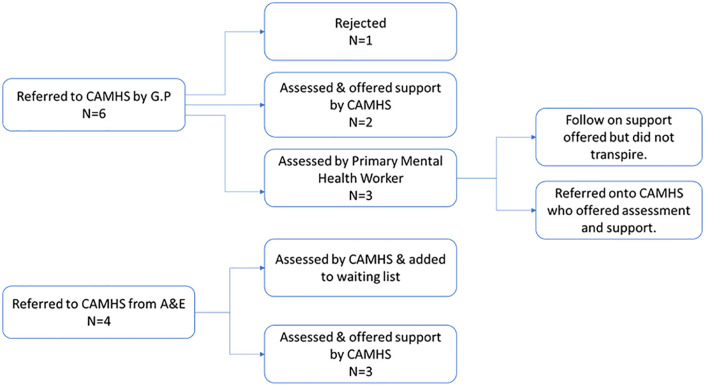
Referral pathways.

Interviews began with each CYP describing (in two instances with support from their parent as they requested for them to be present) their referral process to CAMHS. This is presented in Fig 1 and shows how children who attend Accident and Emergency (A&E) were assessed by a CAMHS worker either before discharge or within 24 hours (n = 4). Children and young people who were referred by their General Practitioner (G.P) (n = 6)were either rejected without an assessment (n = 1), had an appointment with a primary mental health worker before being referred onto CAMHS (n = 3), and n = 2 were assessed and offered immediate access to CAMHS support.

Following a process of iteratively coding the data in Nvivo, and creating concept maps to visualise the data and relationships between codes the following themes, and overarching argument were identified from the stories that were shared (Table 1: Summary of themes).

**Table 1:** Summary of themes.

Substantive theory	Seen but not Heard	CAMHS do not meet the needs of suicidal children and young people even when they are seen.
**Theme 1**	***“Nothing got resolved”***	**This theme explores what happened when they got to CAMHS, and how the children experienced this.**
**Sub-theme 1.1**	***Hoping for help***	**Most of the young people had gone to CAMHS hopeful that it would be a helpful experience.**
**Sub-theme 1.2**	***“Nothing really happened at CAMHS”- frustrated, disappointed and let-down***	**Many of the children reported that attending CAMHS made no difference to them. They did not feel it was beneficial.**
**Sub-theme 1.3**	***Prescribed approach***	**Workers were described as using a clinical approach, which some felt led to them having preconceived ideas about what was wrong.**
**Sub-theme 1.4**	***Generic advice***	**Advice was often found to begeneric, with the same advice imparted to all young people regardless of their individual needs and presentation. This was often experienced as unhelpful and dismissive of the child’s painful situation.**
**Sub-theme 1.5**	***Ready or not: case closed***	**Children/ young people were often discharged without warning or planning, before they were ready.** **They experienced being discharged before they were ready as a rejection/ dismissal and this put them off seeking help from CAMHS in the future.**
**Theme 2**	***“If you had more of a choice…”***	**This theme shows how the children and young people were given little choice: about where they were seen, how they communicated with workers, or parental involvement.**
**Sub-theme 2.1**	***Location, Location***	**Although a few were seen within school many children described having to attend clinical environments at locations that were inaccessible to them without their parents taking them. In site A -CAMHS was too far away for one young person to be able to attend. There were requests to make the location of the appointments more flexible, with the option of a non-clinical environment.**
**Sub-theme 2.2**	***Get in touch …***	**Generally, the children and young people did not feel they would or could contact CAMHS. They would have preferred different means of communication than letters being sent to their home address or being asked to telephone for support if they needed it. Young people reported more positively on services offering text messaging, and more informal communication and appointment systems.**
**Sub-theme 2.3**	***Family matters & Confidentiality***	**Whether parents should know their child was seeing CAMHS, or the extent they should be involved in appointments, was complex and varied depending upon the individual child’s circumstances. The children needed their views about this to be acknowledged from the outset, in order to be able to engage with the service. Confidentiality was important to all the children and young people. This extended to all areas of interaction with CAMHS and wider services.**
**Theme 3**	***The person not the “profession” that helped.***	**There was a wide range of people the children and young people identified as having helped them. But they all shared common characteristics such as being easy to talk to, listening, believing, and being trustworthy, that were prioritised above the person’s position or occupation.**

## Theme 1: “Nothing got resolved”

### Theme 1.1: Hoping for Help

*“Yeah. I did ask for help ‘cause I thought it would, like, help me.”* (Resopndent6)

Despite feeling unsure of what to expect, the children and young people sought support from CAMHS hopeful and with an open mind.

*“I was nervous but glad at the same time to, like, speak to somebody, d’you get me?” (Respondent7)*

*“It was good, I was still nervous and stuff.”* (Respondent8)

Some reported feeling relieved that they were finally able to speak to someone they believed would be able to help. Only one CYP’s testimony did not reflect this. This particular CYP had not wanted to be referred to CAMHS, and shared that had been scared to talk to anyone, fearing parental involvement or hospitalisation.

*“I felt if I said anything or that she’ll tell mum or dad or, like, take me to A&E” (Respondent1)*

*“Confidentiality was vital for this child, and they had yet to be assured that it was safe to open up. Yeah, I understood it’s confidential but… don’t know, I can’t trust anyone.” (Respondent1)*

For other CYP, including those who had been rejected by CAMHS, they had approached CAMHS believing that this the service would provide the support they needed.

### Theme 1.2: “Nothing really happened at CAMHS”- frustrated, disappointed and let-down

Participants described having attended CAMHS hoping it would be helpful, however many reported they were disappointed, as it did not help them address their worries.

*“Nothing really got resolved. I usually just talked about my day. I only went there for like, well, five weeks and then I got, like, told my mood seemed good enough that I could stop going or something like that, …but yeah. […] I felt… honestly, I felt like nothing got resolved, like, nothing, you can’t really get resolved in one session, but like nothing really happened of it.” (Respondent6)*

Accounts highlighted nothing having changed for them following their attendance at CAMH services. Respondent6’s use of the phrase “*nothing got resolved*” points to a quest for solutions that had not been found. They felt they were left no better off than when they started.

*“Not really no, nothing really happened at CAMHS, I only really talked about my day and then five/six weeks later I got sent off and then that was it and I was back to me in my house and all that.” (Respondent6)*

Much of this frustration seemed to be related to participants not feeling supported or understood by their worker. Participants spoke of how they felt their workers didn’t engage relationally with them, or what was going on for them. They spoke about their worries not being taken seriously, dismissed, or missed altogether.

*“It was just similar to speaking to like a parent, I don’t know, it wasn’t really… she basically, cause obviously when I was talking about what happened and that, she basically just said it was probably hormones and I just didn’t think that was very… “(Respondent3)*

One child spoke about how it was only after having engaged with CAMHS on two separate occasions about six months apart that their worker recognised the main underlying issue for them.

*“[CAMHS WORKER] only really properly picked up on it, like, a few weeks ago but I don’t know if she’s really, she’s gone a bit into it but…”(Respondent8)*

Despite hoping they would be allocated someone they could talk to, not all the children found their workers easy to talk to.

*“She wasn’t really the best; she wasn’t really a warm character so it was quite difficult to talk to her.” (Respondent3)*

Not feeling they could talk to their worker, or supported by them to open up, left the children feeling it was all a bit pointless.

*“It was even hard to speak to my parents about it never mind someone that I’d never met before, and I understand she’s a medical person but it was just the way that she was kinda like a bit careless, like, she didn’t really care really, it sounded as if she was just like ‘oh you don’t seem that serious, okay there you go, over’ that was pretty much it. So, I don’t think it helped.” (Respondent9)*

A few children spoke of feeling their workers did not ‘really care’. Although this was also reported in relation to professionals in other roles (such as G. P’s and a primary mental health worker), when it was perceived in their sessions with CAMHS workers, it was experienced as an ultimate disappointment, having believed this person would have been able to help them. For some, this compounded their beliefs that they were not worthy of help or could not be fixed.

*“They all do good and they all try their best but sometimes there’s just people that can’t really be fixed I guess, ‘quote/unquote’.” (Respondent6)*

The child’s further internalisation of suicidal thoughts, and their belief that they could not be helped, an unintended consequence of a failed engagement with CAMHS, highlights a potentially dangerous outcome that could deter future help-seeking.

Only two participants shared having had a positive experience of their worker. One spoke of feeling they could relate to them, and that the worker recognised and understood their difficulties.

*“No, I think it was actually really good, it was very positive. There wasn’t really anything that could’ve improved it really cause I got on really well with the counsellor and we talked about, like, sports and stuff, so at the start off of things he’d start off by just sort of easing me into the conversations by talking about sports, like, the football scores at the weekend and stuff like that, sort of ease me in cause I’d still be anxious to meet, like, even though I’d been going for a while I’d still be anxious the first minutes of the session and then I’d ease back in.” (Respondent10)*

One other child shared having a positive session with a CAMHS worker, but unfortunately, they were transferred to someone else who they didn’t have a connection with.

### Theme 1.3: Prescribed Approach

Several participants described workers using a ‘clinical’ approach in their interactions, which contributed to them not feeling understood or listened. A clinical approach was identified by the children as contributing to them not feeling understood or listened to. This manifested as workers being cold, distant, and applying set ways of working.

*“The way that she treated me was kind of like… careless and kind of like I was not a person but more like clinical if that makes sense, I was more like my circumstance than an actual person going through that. It was quite uncomfortable, like, awkward, I found it quite… I don’t know how to describe it; it was just really… I don’t know, I didn’t find it helpful.” (Respondent9)*

Some of the children reported they felt that their worker wanted to label them. They experienced this as a way of dismissing their worries, providing a medical explanation removed from their social and emotional world.

*“…when I was talking about what happened and that, she basically just said it was probably hormones and I just didn’t think that was very…”(Respondent3)*

*“…with the lady at CAMHS the atmosphere was quite uncomfortable, awkward, I did feel quite intimidated. If I said something and it didn’t fit into kind of what her mindset was or what box she wanted to put me in, she kinda didn’t act like she cared about my certain situations, so with her I kinda felt as if she wanted me to say, like, she wanted me to say certain things for her to be able to, like, tick me off as having depression or tick me off as, yeah, …” (Respondent9)*

By concentrating on symptoms, and potential illness, the focus was shifted from difficulties the children were facing at home or in their personal lives, to what was *wrong* with them. When the dialogue around suicidality was reduced to potential medical conditions, it resulted in the CYP feeling misunderstood. Their own thoughts about what was going on were different to this. However, as a child seeking help from a medical professional, they did not feel able to articulate this.

*“Well I just thought it wasn’t,” (Respondent3)*

*“I could probably more stand up for myself and, like, make it known that maybe it’s not what they think it is. I don’t personally know if it’s one or the other but, like, I would say it’s definitely not stress. It’s gone on for so long.” (Respondent2)*

This dissonance often remained unspoken between the child and the worker but contributed to the children feeling their CAMHS worker did not understand or help them.

### Theme 1.4: Generic advice

Many of the coping strategies suggested to CYP by CAMHS workers could be considered ‘self-help. They described self-harm minimisation advice, distraction and relaxation techniques, as well as mental health mobile phone applications (APP’s) that were recommended. Most CYP reported that they did not find much of this to be helpful, or reflective of what they felt they needed help with.

*“She never really gave me any strategies, she just… the most things she said was, … she always, basically all she said was if you’re feeling that just go, like, go do an activity for, like, 20 minutes, just keep your mind busy. That’s basically what she said.” (Respondent3)*

*“The only thing I remember is the woman asked ‘what are you using to cut?’ and I was like ‘a Stanley knife’ and they’re like ‘don’t do that’ and I was like ‘okay’ and they’re like ‘if you’re going to cut use, like, a razor, like a pencil sharpener razor’. […]” (Respondnet4)*

Some CYP reported that they had never tried to implement the advice or look at the resources that were suggested. Others felt these were things they would have done by themselves anyway. But, in the main there was an incongruence between what the children needed and the generalised advice or standard responses they received.

*“Like, if you’re at a point where you’re low you’re not really going to concentrate and, like, really anxious. I was really anxious to sit and meditate, like, I think that was a weird one.” (Respondent4)*

The children articulated needing different things. For example, one person had tried multiple on-line resources and found some to be helpful for a while, whilst another clearly stated that this would not be of interest to them.

*“It was good up until I kind of… just didn’t see the point in the app because it stopped kind of helping.” (Respondnet2)*

*“I think when I’m at home I just want to be at home, I don’t want it to be, like, part of my therapy or whatever.”* (Respondent10)

Although there were a few positive reports of advice and self-help strategies being given, as in the example above where they found the APPs helpful to begin with, overall, CYP did not provide positive accounts of their usefulness, and perhaps most importantly, their worker did not seem to discussed their acceptability or effectiveness with them directly

### Theme 1.5: Ready or not – Case Closed

Most children reported discharge happened before they were ready, and there were several for whom suicidal ideation continued to be an on-going issue. Far from being a collaborative process and ‘planned for’ event, it was often sudden, seemed to come without warning, and was experienced as a rejection, or dismissal.

*“She was just like ‘oh I think you’ve got better, I think you should just, like, go home, you don’t really need to come back here’ she gave me her phone number.” (Respondent8)*

*“I didn’t really want to see her but she said that basically ‘cause she thought I was fine to be discharged. […] I thought ‘well I’m not’ [laugh].” (Respondent3)*

Some participants expressed they were continuing to self-harm, and/or were thinking about suicide at the time of discharge. A few of the children disclosed they had told their worker they had stopped self-harming or had no plans for suicide to allow the sessions to end as they had not felt they were beneficial.

*“I just said I stopped cutting and she discharged me.*
*[Interviewer: And had you?]*
*For about two weeks.*
*[Interviewer: Right okay. And did you tell her that so that she didn’t come back to see you?]*
*Yeah.*
*[Interviewer: Right okay, and what about the suicidal feelings and stuff, was that something that you were able to be honest about?]*
*No” (Respondent1)*
*“She basically just asked if I’d made plans, like, plans to attempt suicide and well I said no ‘cause I didn’t actually.*
*[Interviewer: So, you didn’t have any plans, but you were still having thoughts.]*
*Yeah.*
*[Interviewer:And were they every day or were they, like…?]*
*I would say pretty much every day.” (Respondent3)*

Others, although accepting of the workers decision, felt hurt and let down by having their case closed before they felt ready.

*“It’s just like demoralising cause I didn’t really feel like, like, I’d improved but I wasn’t, like, better.” (Respondent8)*

Many of the children related that upon discharge they were told to get in touch if they needed to. If they didn’t contact CAMHS within this time it was assumed they were ‘fine’. Most said they wouldn’t have contacted the worker at this point even though their issues remained unresolved. Having experienced rejection and not feeling understood, the sentiment of ‘what would be the point’ was conveyed by the children.

*“Probably not because I didn’t find it helpful. Although maybe if I was quite desperate maybe I would but no I wasn’t in that situation, luckily, luckily I wasn’t in that situation.” (Respondent9)*

*“[Interviewer: Would you ever go back and seek support from mental health services?]*

*Not really, I don’t really know.” (Respondent6)*

Several children were re-referred to CAMHS following a repeat suicide attempt or increased suicidal ideation. Parents often requested the re-referral for them, via the G.P, but one child who didn’t feel able to speak to their parents due to challenging family circumstance’s, spoke about having to find the courage themselves to ask for help a second time.

*“[Interviewer: Was it easier going for help the next time or was it just as difficult?]*

*"I got in easier but asking was harder.” (Respondent8)*

Being discharged and told to get in touch if they needed anything was experienced as a rejection, but also as a communication that they ‘*should*’ be okay. Most interviewees did not feel okay at the point of discharge, but did not challenge this.

## Theme 2: “If you had more of a choice”

### Theme 2.1: Location, Location

Appointments were generally held in hospitals or other clinical locations that the child was invited to attend.

*“It was in a clinic, well was it a clinic, yeah it was in a clinic the first one, but then when I moved to the guy it was in sort of a building just for CAMHS, it was like just a building for CAMHS yeah.” (Respondent10)*

*“I went to the hospital and I saw them, …”(Respondent3)*

CAMHS clinics in site B were not easily accessible by public transport, or near where the CYP lived. This meant they needed to be taken to their appointments, as they would have been otherwise inaccessible. Subsequently they had little choice about informing/ involving parents.

*“My mum had to come cause I was under the age of 16, like, she doesn’t come in but she has to drop me off cause it’s in the middle of nowhere.” (Respondent8)*

*“…yeah he would always take me or my mum would take me in the car” (Respondent9)*

*“My mum took me because at that point I was at [Location] so I had to be, like, driven.” (Respondent3)*

One participant in Site A spoke about how living rurally meant CAMHS was too far away to be an option for them.

*“I’d have to go down to xxxxx for their kinda CAMHS situation which would be, like, a long journey and in there for, like, half an hour kinda meeting and then have to travel back home. It’d be long day.” (Respondent2)*

For those who had seen CAMHS, the space within which the appointments took place were described almost as empty rooms, with a formality to them.

*“Well, l I went in and it was basically just a room with three chairs in it, and then I’d to sit down and I would just being a bit awkward [laugh],...” (Respondent3)*

Although the children spoke about how it was difficult to get to these places without their parents taking them, most seemed to accept without question that this was where the appointments were held. Although they may have preferred for their parents not to be there, many were resigned to the need for parents to know about and transport them to appointments

Not all the young people wanted their parents knowing that they were seeking mental health support, or why. Autonomy and confidentiality were of the utmost importance to most of the young people interviewed. Asking them to attend appointments at hard-to-reach hospital locations with their parents potentially compromised this from the outset.

*“They know ‘cause they had to know but, like, I didn’t really want them to know…”(Respondent8)*

*“…I refused to take my mum and that, cause then they’d find out and I didn’t really want her to find out …” (Respondent2)*

Two children from site B spoke about their worker coming to see them in school. This followed their initial appointment within the hospital setting, and for one child only happened upon their second time of using the service following a re-referral. Appointments taking place in school did not necessarily mean they were in a more positive environment, or easier to attend. The room provided by one school was described as being akin to a disused storage cupboard, and one of the children was so keen to avoid the appointments they deliberately skipped the classes at the times the worker had told them they would come.

*“I didn’t always go to them, but she’ll try and get me out of class so I just maybe missed, don’t go to the class” (Respondent9).*

This CYP’s account stood out from the other interviews as they had not wanted CAMHS support. For the other CYP (Respondent8) being able to see a worker in school had been pivotal in them being able to access support as they had not wanted their parents to know.

*“Yeah, cause it also means my parents, like, I think they know now but they didn’t know when I first went.” (Respondent8).*

Although seeing someone in school was helpful for this CYP, others stated this would not have been their choice, as it would compromise their confidentiality with peers and teachers, highlighting just how important choice of venue was to them.

*“…just a sort of place that you don’t feel judged really, cause sort of in school if you were to talk about it, I don’t know, I feel like I’d be judged because people or teachers would know and I wouldn’t want them to know cause even though they probably wouldn’t be judging me, I’d still be feeling judged. It’s just sort of an environment where you don’t feel judged is really what I feel is the best sort of thing.” (Respondent10)*

The interview data shows the children considered not only the location of the appointment to be important, but the overall environment.

*“…think in a location that’s not in a hospital or something that’s like, just the clinical side of it kinda like makes it a bit iffy and uncomfortable, and as soon as you walk into that environment you’re going to… your mindset’s going to be like ‘oh no take me out of here, I don’t want to speak about how I feel, I don’t want to speak to this new person’, but if you’re in your own house or your own environment you kind of will feel a bit more comfortable speaking to that person. Like, even now I feel more comfortable speaking to you [laugh].” (Respondent9).*

The data suggest that one of the reasons some children found other services more beneficial was in part due to the flexibility and choice they were able to offer about where appointments took place, and there was a more collaborative dialogue around this.

*“I think if you got to choose more where they, like, met you.” (Respondent3)*

Given that even amongst this small sample of children there were a variety of opinions about the best place to meet it would suggest that being given a choice around this would be beneficial.

### Theme 2.2: Get in Touch

Overall, the CYP expressed that they did not feel able or likely to contact CAMHS themselves. They indicated a preference for alternative methods of communication, rather than receiving letters at home or being expected to make phone calls to a CAMHS office asking for support. The CYP were asked about their communication with workers in other services that they identified as having been more helpful. They shared how these workers had often been flexible about where and when they met and had arranged home visits or took them out for a coffee etc. Some had also taken the time to check up on how they were when they had not seen them, or during holiday periods.

*“She was really open. If you had a problem, even if it was like when she was busy doing work, she would stop and she would come and speak to you or she’d take you out of class, take you out in her car and take you for a drive for ten minutes and just get your mind off of it, and then you go back to class and feel better, not fully better but as good as you could which is better than here, …” (Respondent2)*

Feeling their worker was available to them and interested in them helped the child to feel supported and open up, and was markedly different from their earlier descriptions of their interactions with CAMHS.

*“I’ve got [workers] number and she texts me quite frequently, like, ‘hi, how’s things?’ ‘can I come see you?’” (Respondent 5)*

For most of the children being given a choice not just about where they saw their worker but how they would communicate was important.

*“I’d do it not by letter, like, maybe text or something, so it’s, like, confidential there. (YP8)*

*If I could book appointments online, I think, …” (Respondent4)*

When the onus was on the child to telephone the service or worker this was often too difficult for them to do.

*“Well with both the private service and with CAMHS they always gave me, like, numbers and stuff but I never really feel comfortable doing that.” (Respondent 3)*

*“I just find phone calls they give me quite bad anxiety.” (Respondent4)*

Offering a range of means of communication and having the worker lead on this by taking the initiative and contacting the child would better support them to feel they could contact their worker if need be.

### Theme 2.3: Family Matters & Confidentiality

Most of the CYP spoke about how they had found it hard to talk to their parents about their suicidality, or what was going on for them. Some remained certain they did not want their parents to know. The fear of their parents finding out that they were accessing CAMHS support, was a barrier to them seeking help or confiding in anyone.

*“Yeah, so my parents actually didn’t know that I was quite depressed so it was quite a shock to them, …”(Respondent9)*

*“ I never really spoke to my parents about that yeah again you feel like you’re failing them, you didn’t want to worry, it’s not really nice to tell your parents why you’re feeling bad, like, they might feel it’s their fault, that sort of thing.” (Respondent4)*

*“Yeah ‘cause I refused to take my mum and that, cause then they’d find out and I didn’t really want her to find out cause she’s going through a lot with her husband,...” (Respondent2)*

*“ I felt if I said anything or that she’ll tell mum or dad…” (Respondent1)*

*“I think yeah, I kinda wanted it to be confidential rather than telling my parents” (Respondent8)*

These children were often dealing with very complex and challenging family circumstances at home, and yet this was not always recognised by services or practitioners. Through the process of seeking help from CAMHS their parents were informed or became involved without their choosing.

*“They know ‘cause they had to know but, like, I didn’t really want them to know cause my mum has mental health issues, she’s off work just now so I just didn’t really want to worry her too much more. […]” (Respondent8)*

One child had expressed to their G.P that they did not want their parent to know, and yet a letter was sent to their parents. This letter being sent to their home without consent may not only have comprised their confidentiality, but also could have had repercussions for them at home. For some, having their parents present in appointments prevented them from being open or sharing how difficult things were.

*“A bit, well I can’t actually… I think I did go with my dad the first time to the doctors and I think because he was there I didn’t really want to say much, …” (Respondent9)*

*“No cause my mum was there so I couldn’t really say anything.” (Respondent2)*

*“I think because I was with my mum and, I don’t know, just ‘cause I didn’t really feel like I could talk about it.” (Respondent8)*

Although not all the children’s difficulties were located within the home, they wanted to be able to choose what was shared with their parents and have their right to privacy respected. Thus, also acknowledging and creating room to discuss any difficulties there may be in these relationships.

*“If they were more, like, interactive with you. I guess if they spoke to your parents, like, after each session, just had a wee chat with them ‘cause I used to be like let go and that was it.”* (Respondent3)

A few reported they had had positive experiences of primary mental health workers supporting them to share information agreed in advance with parents.

*“I think the first lady that I spoke to I found her really, like, she was listening to me and she was speaking to me about how I could, like, communicate and stuff with my parents and address stuff, like, feel comfortable speaking to them, and she brought my dad in with permission and she kind of explained for me because I was really upset at that point, I mean, when kinda told my dad ‘cause I couldn’t, but she was really, really, she was really good at doing that.”* (Respondent9)

There was also one positive example of a CAMHS worker supporting a child to share agreed information with their parent following the appointment.

*“So I always don’t like my mum being in the session if I’m speaking to someone ‘cause she always tries to sort of take over the conversations, cause she’s a chatty person and so he, ‘cause we’d talked about it a bit, and he’d to get my mum to come in to talk about what we did and stuff, he didn’t sort of like stop her but he would sort of try to tell her, well not tell her but talk so she wouldn’t be able to talk cause he knew I didn’t like that. So, he did that well, but he did talk to my mum and tell mum the stuff that I wanted her to hear, not the stuff that I didn’t want her to hear well.” (*Respondent*10)*

Being supported to talk to their parents was a key factor in what made the intervention beneficial for these children. However, this would not have been what all the CYP would have wanted, or found helpful, and some would have rather their parents were not involved at all.

The children’s family situations and relationships were complex and diverse, and yet this was not always felt to be understood or reflected in the approach adopted by CAMHS, or the services making referrals to CAMHS. This is bound up within issues of confidentiality that was not always afforded to these children: their parents were informed about the referral being made to CAMHS, parents were invited to attend appointments with their child without their child being consulted, and children often needed to rely upon their parents to take them to appointments due to their geographical location. Overall, regardless of their situation confidentiality was of great importance to the children interviewed.

## Theme 3: The person not the profession that helped

*“I’d make it, like, completely confidential…” (*Respondent 8)

When the participants were asked if there was anyone they would go to for help, or if there was anyone who they felt had been able to help them, it was clear they identified the person’s personal characteristics and how they made them feel beyond their job role or any treatment approach. For most, the individual had not been a CAMHS worker but included a family member, friend, support worker, social worker, or primary mental health worker.

*“Right now I’d go to my dad or my pals online. They’re, like, the only people I can really confidently speak to about this stuff.” (Respondent6)*

*“… my best friend […] she’s been more helpful than the mental health worker that I’ve got, although she doesn’t know anything about mental health…, but she’s just been there through everything with me and she’s stood by me even when we weren’t close she would still stand by me.”(*Respondent2)

Friends and family were repeatedly identified as the key confidantes, but when it was a support worker/social worker/primary mental health worker who had helped, the children spoke about how they made them feel relaxed, and that they could talk to them.

*“Yeah. Her approach was less clinical if that makes sense, she was less… I don’t know how to put it, she was not trying to put me in some kind of category or making me say certain things, that it wasn’t as intimidating with her, whereas with the lady at CAMHS the atmosphere was quite uncomfortable, awkward,…”* (Respondent9)

*“I had a social worker called, xxxx was her name, and she was helping me at school, like, she’d come and see me every week and we’d just talk about, like, how I felt and things like that and she was really good,…* […] *Yeah, I’d quite often speak about that* [suicide and self-harm] *with XXXX but we didn’t really do much work on it, like, I don’t know what type of work you’d do but we didn’t really do much work about it, we’d just talk about how I was feeling and if I was feeling better or if I was feeling worse, it was just that type of thing.”* (Respondent5)

The person they felt had helped them was identified as trustworthy, and the children felt they could confide in them. They were *“warm”, “open”, “friendly”* and *“nice”*. They were interested, interested in the child and what was going on for them. They showed compassion and adopted a non-clinical approach. Perhaps above everything they listened.

*“I think the first lady that I spoke to I found her really, like, she was listening to me…”* (Respondnet 9)

*“Well, he was just more warm, like, and also he didn’t go full in, like, first day, you know, cause obviously he just warmed into it instead of all of a sudden being, like, really heavy.”* (Respondent3)

The only CYP who reported that attending CAMHS had been beneficial equated this to having had a positive relationship with their worker and being able to get along with them.

*“… I got on really well with the counsellor and we talked about, like, sports and stuff, so at the start off of things he’d start off by just sort of easing me into the conversations by talking about sports, like, the football scores at the weekend and stuff like that, …”(*Respondent10)

The relationship the worker had with the child outweighed other factors such as their job role. As can be identified in many of the quotes above, the children and young people valued people engaging with them on their level and adopting a non-clinical approach. When asked directly what they would want from a worker their responses conveyed a need for the person to be approachable.

*“I’d make it, like, completely confidential and make sure nice people are employed as well. […] Like, friendly and kind I guess. Don’t know, I’m trying to think, whatever you’d put with a nice person.” (*Respondent9)

Not only did the worker need to be “nice”, but to show that they cared. That they cared about the young person and what was going on for them.

*“Someone to care because not a lot of people do, even though they say they do, like, …to actually understand that people. I get a lot of people saying ‘I understand how you feel’ but they don’t and it’s like, don’t know how to, like, respond to them sometimes.” (*Respondent*2)*

### Substantive theory: Seen but not Heard

Overall CAMHS did not meet the needs or expectations of most of the children and young people interviewed for this study. Only one young person spoke of having been allocated a CAMHS worker who ‘got’ them and feeling supported with the things they were struggling most with, including talking to their parent. Most children in this study and were either rejected (at the point of referral) without being seen by CAMHS, or when they were seen, did not feel their worker really saw what they were struggling with or heard what was going on for them.

The themes we have presented show that the children understandably faced barriers in being able to speak openly about what was troubling them, and yet the sessions they described did not support their engagement. They remained clinical and prescriptive, without making space to get to know or listen to what the child or young person needed. The young people did not identify the standard self-help advice they received as beneficial. Discharged before they were ready, young people were left feeling let down by the service they had hoped would help them.

Our over-arching interpretation, and argument is that although the children were processed by CAMHS, in the main those that were seen did not feel heard, particularly in relation to not having their suicidal thoughts acknowledged but instead having their issues presented as other conditions (stress depression). This is a unique finding in relation to suicidal children and young people’s experiences of CAMHS. The children and young people wanted and needed different things from the service, yet there was little flexibility or choice offered. The lack of a collaborative and supportive relationship left the children feeling their needs had not been met by CAMHS.

## Discussion

Overall, the findings from this study are supported by other qualitative studies exploring the views of CYP experiencing suicidality [37,47,48], as well as those of mental health support services for CYP more generally [49,50]. However, very few other studies specifically included participants who were suicidal, or under 16 years, therefore the findings of this study are indeed a novel contribution. For example, recent research in the UK about CYP mental health services more generally, has also found that children and young people prefer community-based services, and non-clinical approaches [10,11], and suggest non-statutory organisations are more agile, and better able to provide the type of flexible support they prefer [11]. Similarly, the findings that children experiencing suicidal thoughts/behaviours do not feel listened to or taken seriously by mental health practitioners, and do not want prescribed clinical models of care, have also been previously reported [48,49,51]. However, what is unique to this study is the negating of suicidal thoughts/behaviours or their translation to ‘other’ conditions that are more in line with clinical conditions such as depression or stress that can be managed with prescribed approaches. The feelings of not being listened to, therefore, are specifically in relation to their suicidal thoughts and behaviours in the eyes of these young people. This can have more significant impact and consequences than for other young people attending CAMHS more generally.

Assurance of confidentiality was deemed to be of paramount importance, and for some CYP it was a crucial factor in determining their engagement with CAMHS. Notably, some participants identified existing CAMHS practices that compromise confidentiality beyond the initial referral made by their G.P, such as routinely sending mail to their family home, including direct correspondence to their parents, arranging appointments at locations that are inaccessible without their parents transporting them, or scheduling appointments within the school setting. It is well documented that children are less likely to seek help with sensitive issues unless they can be assured it is confidential [52]. Other studies have also shown that children often feel CAMHS do not provide enough information about their rights to confidentiality and what information would be shared for them to have confidence using their services [15].

Qualitative research conducted in Canada [50,53] and other European countries [54], as well as the UK [55,56] all present similar findings in relation to CYP views and experiences of mental health services: they often feel their needs are not met and would prioritise the relationship with the worker over seeing a particular professional.

The finding that it was the person and not their profession that made the difference to the CYP, and the importance of the relationship between the practitioner and the child has also been identified in other child and adolescent mental health research [57,58]. The power of the therapeutic relationship has long been recognised within the field of adult mental health. The ‘Dodo Effect’ (a term initially coined by Rosenzweig, 1936 [59]) refers to the suggestion that all psychotherapy treatments are as good as one another. Smith and Glass, 1977 [60] showed that whilst many psychotherapy treatments had evidence of effectiveness there was no difference in effect between the types of psychotherapy. However, none are found to be beneficial unless the practitioner has a positive rapport, and engagement with their patient or client. Some studies have shown that the difference in effectiveness is due to the individual therapist delivering treatment [61]. As stated by Richard P. Bentall, 2010 [61], the work of Carl Rodgers has been instrumental in understanding the nature and importance of the therapeutic relationship in what has been termed ‘the therapeutic alliance’ [62].

Despite the recognition of the importance of the relationship in delivering mental health support, there has been little attention paid to measuring the therapeutic alliance or connection between practitioners delivering child mental health interventions and the child/young person [63]. The focus continues to be upon developing processes and prescribed approaches, neither of which are reported to be helpful to children who are considering or have attempted suicide. As noted above, pursuing such an approach results in children and young people who are experiencing suicidal thoughts and behaviours experiencing this as negating or translating their experiences into something else.

Children and young people who experience suicidality are not a homogenous group. Research has found that there are often multiple and often overlapping causes and risk factors for suicide in children and young people, such as childhood adversity, trauma, witnessing domestic violence, parental separation, bereavement, bullying, and social isolation [51,64]. Although not limited to certain groups, some have been identified as at greater risk, including LGBTQ + , and care experienced young people, as well as those with neurodiverse conditions such as autism, and those living in rural areas. Prescribed clinical approaches and short-term interventions do not provide or create the space for exploration of the social context and complex underlying issues that CYP experiencing suicidality are facing [51].

Although children and young people are more involved in mental health research studies [65,66], and are now beginning to be consulted by service providers [15,67] and policy makers [68–70], there remains a barrier in relation to implementing their recommendations. A systematic review in this area found there to be no studies that demonstrated a change in practice following input on service delivery by CYP into adolescent mental health services [57]. Although including children and young people’s views in service design and delivery is considered to be best practice [70–72], evidence of tangible impact remains elusive.

Qualitative studies with children and young people who have been or are suicidal, generally have small samples, and limited generalisability. It could be that qualitative evidence is not considered ‘good enough’ to be used to inform practice guidelines which tend to rely on evidence from RCT’s [58,73], reflecting a bias towards quantitative research identified in suicide research more generally [74].

There is an urgent need to align the views and experiences of CYP with the mental health support services provided, to ensure that they are beneficial. If we continue to provide services that are not working (from the perspective of CYP) we risk compounding rather than helping CYP with their suicidality, and deterring them from future help-seeking.

### Strengths & limitations

To our knowledge this study is the first to present the perspective of children and young people accessing CAMHS for reasons of suicidality, about their care journey and the support they receive. It offers valuable insight into their views and experiences, providing new knowledge about why CAMHS does not seem to be helpful for many children and young people experiencing suicidality, as well as suggestions of things they would like from mental health support services.

Although there is some overlap with research findings pertaining to CYP’s perceptions and experiences of CAMHS more generally, this study is unique in that the sample population all had experience of suicidality and were referred to CAMHS following a suicidal crisis. Understanding their specific needs, and experiences can undoubtedly support developing more responsive and acceptable services that can better address the rising numbers of children and young people who die by suicide.

The sample population was relatively small, making it hard to *generalise* from the findings, and also demarcate between those who were seen by CAMHS or rejected/on the waiting list for analysis purposes. However, recruitment to qualitative studies about childhood suicidality is particularly challenging given the vulnerability of participants and sensitivity of the topic, and we faced the additional barriers that COVID 19 lockdown restrictions imposed. There are arguments for the use of small samples, especially with hard-to-reach populations such as this [75]. Recognition of the richness and quality of the data is suggested to be more important in qualitative studies than the number of people participating [31], and arguably trying to treat qualitative research studies in the same way as we do quantitative studies is highly inappropriate and misses the value and contribution of qualitative research [76]. Recruiting participants directly from the sites where the retrospective cohort study was conducted [8] meant that although it could be suggested the sample is not representative of all children and young people, they were *‘informationally representative’* [31] of the experience of children and parents referred to CAMHS in these areas for reasons of suicidality. Although the sample size was relatively small, the data was rich and the rigorous analysis employed ascertained validity.

A further limitation is a lack of diversity in the sample, participants were predominantly white British females. This is perhaps unsurprising given the lack of diversity in the population within the localities where the studies took place and that 96% of the Scottish Population identify as white [77], or indeed the lack of data available regarding the numbers of children from minority ethnic background referred to CAMHS for support in Scotland [78]. However, the convenience sampling approach which was necessary to satisfy General Data Protection Regulation meant a targeted approach to recruitment which may have allowed purposeful recruitment of participants from ethnic minority backgrounds, was not possible. Unfortunately, lack of diversity in small samples such as this is not unusual in suicide studies and it is important to acknowledge where gaps in evidence remain.

Additionally, we were unable to recruit any participants under the age of 12 despite one third of eligible participants who were invited to participate being under age 12 [8]. This could be a limitation of the recruitment strategy, which necessitated invitations were sent via post from the Child and Adolescent Mental Health Services. There is no means to establish that children under age 12 received these invitations, as parents/carers could have with-held them, and/or may not have been supportive of their participation. Direct engagement with young people and families may have supported improved recruitment overall, however there remains a gap in research of the views and experiences of younger children referred to CAMHS for reasons of suicidality.

Although this study was only conducted in two sites in Scotland, we suggest the experiences presented here are not uncommon in other parts of the United Kingdom [10] or in other nations where clinical approaches to mental health support for children and young people may hinder their accessibility and helpfulness to those using the service. However, further research is urgently needed to identify and explore the views and experiences of children and young people from marginalised and ethnic minority communities.

## Conclusions

This study aimed to explore children’s experiences of being referred to CAMHS for suicidality and to present their care journey from their perspective. Importantly, it shows that the problem with CAMHS is not solely that they do not have the resources to meet demand for their services, but that even when they are seen, children and young people who present as suicidal do not feel heard, and the model of care provided does not meet their needs. The manner in which they do not feel heard, such as feeling their suicidal thoughts and behaviours being negated or translated into ‘other conditions’ has also been highlighted by this study. They would prefer a more flexible, less clinical approach that respected their right to confidentiality and afforded them the time and space they need to explore the underlying and contextual issues contributing to their suicidal thoughts and behaviours. By failing to listen to CYP experiencing suicidality, or provide them with acceptable, timely, mental health support that is appropriate to their needs, there is a much higher risk of them being deterred from future help-seeking, and going on to end their lives.

This finding has implications for policy makers and service providers as the CYP in this study express that the model of care and approach they need, and want, is not congruent with CAMHS. Further research is needed to identify together with children and young people, a model of care that would be more acceptable, and beneficial to them.

## Supporting information

S1 File Interview topic guides. (PDF)
