# Supplementary material for: Seen but not heard: A qualitative interview study exploring the views and experiences of children and young people referred to Child and Adolescent Mental Health Services for support with suicidality
Source: PLOS Ment Health. 2026 Jun 30;3(6):e0000539. doi: 10.1371/journal.pmen.0000539 (PMC13318000; doi:10.1371/journal.pmen.0000539)
Supplement: S1 File — (PDF) [file pmen.0000539.s001.pdf]

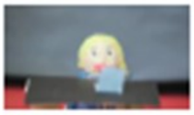

Interview Topic Guide: children / young people

Interview Focus

The main purpose of interviewing children who were referred to CAMHS for reasons of suicidality is to capture their experience of what happened after this referral, in their own words, and from their perspective. The interview will encourage them to tell their story chronologically from the point of referral. Topics of interest include:

- When did they (or someone else) begin to think that something was wrong and that they may need help? What happened next?
- The referral process (Who referred them? Did they know / consent? Did they wait a long time to be seen?)
- The assessment (Did they go to CAMHS for an assessment? If not, why not? If they did what was this like?)
- What happened next? (Did CAMHS offer them a service? Did they get signposted on to another service? Etc.) How did they feel about this?
- What helped?
- Was there anything that didn't help or was difficult?
- What would have been helpful or what would make things better?
- How do they feel about their experience overall? How are they feeling now?

Supporting structure

- Many older children (11 years +) will not appreciate being referred to as children and the researcher will use the term young people when and where appropriate.
- Before the interview starts the researcher will spend time establishing informed consent, using the animation, information leaflet and talking mats.

- They will introduce the topic of their conversation, and stress that they are interested to hear about the child's story in their own words. They will explain that there are no right or wrong answers, and stress if they want to take a break at any time they can, and if they do no not want to answer a question they do not have to, and that will be okay.
- The researcher will emphasise they want to know about the help that the child received from CAMHS or other services, and not with what caused their suicidal thoughts / behaviors'.
- The interviewer will support the child to identify a person that they might talk to after the interview if they feel worried or upset.
- The researcher will offer the child a range of materials that may help them to relax and engage in the conversation (art materials such as colouring sheets, pens and pencils, play-doh, and fidget toys such as tangle sticks).
- The researcher has many years of experience of working with vulnerable children and will ask open ended questions supporting a conversation around the topic rather than presenting a list of questions. The researcher will also encourage the child to keep conversation to the topic area and maintain safe boundaries.
- When the interview is finished the interviewer will ask the child how they feel about what they have spoken about and again ask them to identify the person they might speak with should they be worried or upset. They will thank them by offering a voucher, and a tailored "Thank You" pack with contact numbers etc. of local resources. The researcher will also ask them if they wish to receive a summary of the main findings once the project is finished.

Distress / disclosure

- If the participant exhibits overwhelming distress OR makes a disclosure indicating they are a risk to themselves then the researcher will stop the interview and follow the distress protocol for children and young people version 1.
- If the participant makes a disclosure that they or another child under 18yrs of age is at risk of harm then this information will be shared with the most appropriate agency – social work / police. The child / young person will be made aware by the researcher that they intend to do this.
